# A feasibility study of a preventative, transdiagnostic intervention for mental health problems in adolescence: building resilience through socioemotional training (ReSET)

**DOI:** 10.1186/s13034-025-00870-z

**Published:** 2025-03-22

**Authors:** Alex Lloyd, Roslyn Law, Nick Midgley, Tom Wu, Laura Lucas, Erin Atkinson, Nikolaus Steinbeis, Peter Martin, René Veenstra, Jaime Smith, Lili Ly, Geoffrey Bird, Jennifer Murphy, David Plans, Marcus Munafò, Ian Penton-Voak, Jessica Deighton, Kathleen Richards, Mya Richards, Pasco Fearon, Essi Viding

**Affiliations:** 1 Clinical, Educational and Health Psychology, Psychology and Language Sciences, University College London, London, UK; 2 Anna Freud National Centre for Children and Families, London, UK; 3 Applied Health Research Institute of Epidemiology & Health, University College London, London, UK; 4 Department of Sociology, University of Groningen, Groningen, Netherlands; 5 Department of Experimental Psychology, University of Oxford, Oxford, UK; 6 Social, Genetic and Developmental Psychiatry Centre, Institute of Psychiatry, Psychology and Neuroscience, King’s College London, London, UK; 7 School of Psychology, University of Surrey, Guildford, UK; 8 Department of Psychology, Royal Holloway, University of London, Egham, UK; 9 MRC Integrative Epidemiology Unit, Bristol Medical School, University of Bristol, University of Bristol, Bristol, UK; 10 School of Psychological Science, University of Bristol, Bristol, UK; 11 NIHR Biomedical Research Centre at the University Hospitals Bristol NHS Foundation Trust, Bristol, UK; 12 Centre for Family Research, Department of Psychology, University of Cambridge, Cambridge, UK

**Keywords:** Transdiagnostic, Psychopathology, Adolescence, Feasibility, Social relationships, Emotion processing

## Abstract

**Background:**

Adolescence is a developmental period during which an estimated 75% of mental health problems emerge (Solmi et al. in Mol Psychiat 27:281–295, 2022). This paper reports a feasibility study of a novel indicated, preventative, transdiagnostic, school-based intervention: Building Resilience Through Socioemotional Training (ReSET). The intervention addresses two domains thought to be causally related to mental health problems during adolescence: social relationships and emotion processing. Social relationships were targeted using principles from interpersonal psychotherapy, while emotion processing was targeted using cognitive-emotional training focused on three areas of emotion processing: Emotion perception, emotion regulation and interoception. The aims of this feasibility study were to (i) assess the acceptability of integrating group-based psychotherapy with individual cognitive-emotional training, (ii) evaluate the feasibility of our recruitment measures, and (iii) assess the feasibility of delivering our research measures.

**Methods:**

The feasibility study involved 41 adolescents, aged 12–14, who were randomly assigned to receive the ReSET intervention or their school’s usual mental health and wellbeing provision.

**Results:**

Qualitative data from intervention participants suggested the programme was experienced as a cohesive intervention, with participants able to draw on a combination of skills. Further, the cognitive-training tasks were received positively (with the exception of the interoception training task). The recruitment and research measures were successfully delivered in the school-based setting, with 97.5% retention of participants from baseline to post-intervention assessment. Qualitative data was overwhelmingly positive regarding the benefits to participants who had completed the intervention. Moreover, there was only limited data missingness.

**Conclusions:**

We conclude that a trial of the ReSET intervention in a school setting is feasible. We discuss the implications of the feasibility study with regard to optimising school-based interventions and adaptations made in preparation for a full-scale randomised controlled trial, now underway.

**Supplementary Information:**

The online version contains supplementary material available at 10.1186/s13034-025-00870-z.

## Background

It is estimated that 75% of mental health problems emerge during adolescence [41, 58]. In response to the individual and societal burden of mental health problems during this developmental period, efforts should be made to prevent the emergence or worsening of mental health problems during adolescence before they become entrenched. To this end, we have developed a novel preventative, indicated (i.e., selective, non-universal), school-based intervention to address mental health problems across a range of diagnostic categories: Developing resilience through socioemotional training (the ReSET programme). This study examines whether the novel intervention was acceptable to young people, as well as whether the delivery of the programme was feasible in terms of being able to recruit a sufficient number of young people to the study. In addition, we sought to examine the feasibility of evaluating ReSET through a trial, by assessing the success of the identification/recruitment procedures, the process of allocating to treatment and delivery of the research measures.

Mental health problems in young people are associated with significant negative consequences across the lifespan. For example, adolescents who experience mental health problems are more likely to experience chronic and severe mental health problems in adulthood [54], experience unemployment [4], and have a reduced life expectancy [29] compared to their peers who do not experience mental health problems. Interventions to address mental health problems are therefore vital to improve outcomes for individuals and can benefit society more widely, such as through reducing healthcare costs [55]. While a number of interventions are delivered within mental health services (e.g., Child and Adolescent Mental Health Services,CAMHS), there are significant numbers of young people who are unable to access treatment because demand exceeds the capacity of CAMHS services [57]. Furthermore, ideally, we would like to reach young people before their mental health problems escalate and become chronic. It is therefore important to develop preventative interventions that can improve mental health outcomes for those at risk and that are delivered in settings that are accessible to young people, such as their school [37]. However, efforts to implement school-based interventions have not always been successful in improving mental health outcomes. For example, several recent school-based trials have demonstrated null or harmful effects for adolescents participating in mindfulness [34] and CBT-informed interventions [2].

To overcome limitations in the provision of mental health care, as well as limitations of existing interventions, we have developed a novel school-based intervention: the ReSET programme. The ReSET programme has been developed as a transdiagnostic intervention, meaning that it addresses common mechanisms presumed to be implicated in a range of mental health problems, rather than single diagnostic categories [11]. Transdiagnostic approaches have a distinct advantage in the context of indicated prevention interventions, as they focus on domains that have the potential to influence a number of different mental health outcomes. This is critical because we know that it is challenging to predict the precise developmental course of elevated mental health problems over development [9, 60], which motivates the need for interventions that are effective at preventing the onset or worsening of mental health problems for adolescents experiencing some level of symptomatology. By addressing common mechanisms involved in a range of mental health outcomes, transdiagnostic indicated prevention programmes, such as ReSET, have the potential to benefit the greatest number of young people and prevent the escalation of mental health problems during adolescence for those at risk.

The two transdiagnostic risk factors that ReSET focuses on are social relationships and emotion processing. Both social relationships and emotion processing have been demonstrated as risk factors for a range of mental health problems in adolescence [38], indicating they are transdiagnostic risk factors for psychopathology. While social relationships and emotion processing abilities have previously been the target of mental health interventions for adolescents, such interventions have focused on *either* social relationships *or* on cognitive training of emotion processing, not both (e.g., [16, 69]). Addressing social relationships and emotion processing abilities in combination may have particular advantages over examining these processes in isolation as there is evidence that these processes have a bidirectional relationship. For example, positive social relationships can provide a buffer against negative emotions [36]. Further, adaptive emotion processing can mitigate the effects of negative social experiences, such as bullying [63]. Therefore, an intervention that addresses these processes in combination has the potential to provide efficacious treatment across a range of mental health problems.

To address social mechanisms implicated in poor mental health, the ReSET intervention draws on strategies from Interpersonal Psychotherapy-Adolescent Skills Training (IPT-AST), an intervention initially designed to prevent adolescent depression [69]. The 8-week intervention provides psychoeducation to young people about effective ways of communicating to mitigate interpersonal conflicts, navigate significant changes and counteract social isolation using ‘communication strategies’ [70]. During the intervention, adolescents are provided with opportunities to roleplay interpersonal scenarios and practice communication strategies to address unmet needs and resolve conflicts [71]. The intervention takes a group-based approach to the prevention of depression, with adolescents discussing possible resolutions with their peers encouraging group-based problem solving [69]. Mid-way through the intervention, parents and carers are invited to a meeting with the facilitator and the young person to discuss strategies taught during the intervention. As a transdiagnostic programme, ReSET broadens this approach beyond the prevention of depression, drawing on key principles from IPT-AST that are likely relevant to interpersonal problems linked to a range of circumstances and presenting difficulties.

In addition to principles from IPT-AST, ReSET draws on established cognitive-emotional training programmes in cognitive neuroscience that target mechanisms implicated in psychopathology. The three mechanisms we focused on are: emotion perception, emotion regulation and interoception. Here, emotion perception refers to an individual’s bias to perceive others’ emotions as friendly or hostile. The training is calibrated to each individual’s ‘balance point’, or the point at which they perceive faces as hostile, rather than friendly [47]. The training protocol aims to shift the individual’s balance point such that they perceive an increasing number of faces as friendly. Emotion perception training has been deployed successfully in the past to shift adolescents’ balance point and improve mental health outcomes (e.g., [35]). Further, we trained participants to utilize emotion regulation strategies that improve mental health outcomes, specifically teaching participants to use distancing (i.e., imagining oneself in the future) and reinterpreting (i.e., considering aspects of the scenario that may not be as negative [25]. Emotion regulation training can lead to improvements to mental health in adolescent populations [74]. Finally, interoceptive ability is trained using a modified version of the Phase Adjustment Task (PAT; [49]). In this task, a participant’s heart rate is recorded and a tone is played out of synchronicity with their heart rate. Participants adjusted a dial such that their heart rate and the tone align. As the starting phase is random across trials, the consistency of the participant’s response is taken to reflect their accuracy. In the training task, participants are provided with feedback to improve heartbeat-tone synchronicity judgements. Previous studies have demonstrated that interoception training can reduce symptoms of anxiety in adults with autism [50], though to date no research has examined whether interoceptive accuracy can be trained in adolescence. The ReSET programme targets these three cognitive emotion processing abilities implicated in mental health outcomes and test whether training these domains, when delivered alongside components of IPT-AST, lead to improvements in mental health symptoms.

However, there is an outstanding question regarding whether the integration of techniques from IPT-AST and the cognitive-emotional training tasks is feasible and acceptable to young people, particularly as the two approaches originate from different disciplines. The novel integration of these established techniques may reduce their individual efficacy (e.g., [74, 47, 69]), as it requires adaptation of established protocols (e.g., [69]) to ensure the intervention is experienced as a cohesive package. To overcome this potential issue, the group-based programme is designed to explicitly include content that provides a rationale for the important connections between one’s emotional responses and one’s experiences in important relationships, and how they relate to each other, to give participants a clear ‘storyline’ about what ReSET is and why it might be helpful for tackling important issues in young people’s lives. Nevertheless, it is important to examine whether the programme is acceptable to young people and whether there are indications that participants find this hybrid approach beneficial.

A potential advantage of delivering mental health interventions in schools is that such interventions can overcome the barriers experienced by some young people in seeking mental health support. For example, travelling to sites to receive specialized support can act as a barrier to receiving mental health support [22, 39], and typically exacerbates existing inequalities in accessing and using mental health service [28]. Developing ReSET for school-based delivery provides an opportunity to offer an equitable and scalable mental health intervention for adolescents. However, integrating mental health interventions into schools is challenging [39]. Staff at these institutions often have limited resources to support the integration of novel initiatives into the school’s routine and therefore implementation requires voluntary engagement by individual staff members. It is necessary to ensure that before undertaking a full scale RCT, the feasibility and acceptability of the intervention and the required research procedures to evaluate it within these settings are established.

We have designed the ReSET programme as an indicated, preventative intervention. Unlike universal preventative interventions, which are delivered to all participants regardless of their presentation, the ReSET programme is delivered only to adolescents who are showing elevated mental health symptomatology [12]. Delivery of an indicated intervention requires several stages of recruitment that can affect the overall sample size, such as initial screening, recruitment of eligible participants into the trial, and finally retention in both the intervention and control arms throughout the study. Due to the multi-stage recruitment and data collection for indicated interventions, it is important to investigate the feasibility of delivering these methods in a school setting.

The aims of this study were to evaluate the acceptability of a research trial of the ReSET programme, as well as the feasibility of delivering the recruitment and research measures in a school-based setting. The first aim was to examine the programme acceptability: whether the content drawn from IPT-AST and cognitive neuroscience could be feasibly integrated into a cohesive intervention rather than being perceived as disconnected topics [37]. Specifically, we aimed to examine how these app-based activities could be integrated into a group format which is primarily about communication, interaction and group problem solving. Relatedly, the cognitive-emotional training tasks used in the intervention have been shown to be effective in individual settings (i.e., individual’s behavior changes in line with the predicted effect of each task), yet the group dynamic may compromise adherence to the protocols. The second aim was to assess the feasibility of recruitment and retention [65], specifically: (i) whether our screening measure (described below) could identify a sufficient number of pupils that were eligible to take part in the intervention and control arms of the study, (ii) whether we could gain consent from a sufficient number of these eligible pupils to enroll in the intervention and control arms, and (iii) whether we could retain both intervention and control group participants throughout the duration of the study, which was the same protocol as the main trial but without a 1-year follow-up of participants recruited to the intervention and control arms [65]. Finally, the third aim was to examine the feasibility of delivering the research measures for our baseline and follow-up assessments in a school-based setting while mitigating data loss.

## Methods

### Participants

Participants (N = 41; 54% female; diverse ethnicities—see Table 1) were from two mainstream secondary schools in East London (M_baseline_ = 12 years, SD_baseline_ = 0.47, Range_baseline_= 2). In the two schools that participated in the feasibility study, pupils were screened using a questionnaire comprising of the Strengths and Difficulties Questionnaire (SDQ; [24]) and Me and My Feelings Questionnaire [13]. For the purposes of the feasibility study, we did not enact the randomization schedule planned in the main trial, in which participants are randomized at the school-year level [65]. This decision was made because one of the schools participating in the feasibility study had agreed in principle to take part in the main trial, and therefore we did not screen pupils from year groups we would recruit from for the main trial to avoid contamination effects. Therefore, in School 1, only pupils in Year 9 were screened and randomization occurred at the individual level within the year group. In School 2, the main trial’s intended randomization schedule was used: pupils in Years 8 and 9 were screened and randomization occurred at the year group level such that pupils from one year group were assigned to the intervention group and pupils from the other year group were assigned to the control group.

**Table 1:** Participants’ ethnicity in the feasibility study

Ethnicity	N (Percentage of participants)
English/Welsh/Scottish/Northern Irish/British	5 (12.5%)
Pakistani	5 (12.5%)
Bangladeshi	3 (7.5%)
Sri Lankan	1 (2.5%)
Caribbean	1 (2.5%)
Somali	1 (2.5%)
Arab	2 (5%)
Polish	1 (2.5%)
Irish	1 (2.5%)
Romanian	1 (2.5%)
Lithuanian	1 (2.5%)
British and Romanian	1 (2.5%)
Albanian	1 (2.5%)
White and Asian	1 (2.5%)
White and Black Caribbean	1 (2.5%)
African-Indian	1 (2.5%)
Indian	4 (10%)
No response	9 (22.5%)

After screening, pupils were invited to participate in the ReSET intervention if they scored above a threshold of lower wellbeing. Specifically, eligibility was defined as having a self-reported Total Difficulties score above 15 on the SDQ. We utilized the SDQ when deriving this threshold as this measure had nationally representative data to determine a score reflecting the top 25% of scores for adolescents this age, whereas these data were not available for the Me and My Feelings Questionnaire. Specifically, this threshold was determined using data drawn from the Understanding Society panel study (see [62]), using the 75th percentile of the Total Difficulties score among 2,100 UK adolescents between ages 10 and 15. The schools recruited for this study were diverse on a measure of socioeconomic background, free school meal eligibility, with School 1 having 25.4% of pupils eligible and School 2 having 13.1% of pupils eligible.

### Procedure

Prior to the screening questionnaire, both schools sent out consent forms to parents/carers of all Year 9 pupils in School 1 and all pupils in Years 8 and 9 in School 2. These forms asked parents and carers to indicate if they did not wish their child to complete the screening questionnaire (i.e., we used an opt-out procedure). A total of 11 parents indicated they did not wish for their child to complete the screening questionnaire (six from School 1 and five from School 2). Pupils who were not opted out by their parents were then administered the screening assessment, for which opt-in consent from the pupils was required. This screening assessment was used to identify adolescents with elevated levels of mental health symptomology. Eligible students identified from this questionnaire were then invited to take part in the study, which required consent from both parents and students.

Once consent to take part in the research assessments had been received for all 20 pupils at each school (10 in the intervention arm and 10 in the control arm in each school), these pupils were invited to complete the assessment battery (see Table 2). Assessments were completed in groups of 5 or 10 pupils from the same year group. Group size was dependent on the room available at the school. For a small number of pupils (N = 3), part of the assessment battery was completed individually with a researcher present due to clashes with their school schedule. Once all pupils had completed the assessment measures, participants assigned to the intervention completed the 8-week programme, whereas control participants completed their school schedule as usual. Finally, once the intervention had been completed, individuals in both the intervention and control arms of the study completed the assessment battery once more. Researchers delivering the assessments were blind to the allocation of participants at the pre-assessment timepoint, though were unblinded at the post-assessment timepoint. Participants were remunerated £15 for each assessment session (£30 in total across both assessment timepoints), though no payment was provided for intervention sessions. After all assessments had been completed, a subsample of intervention participants was invited to take part in an interview about their experiences of the group. These interviews were conducted at the participants’ schools and took approximately one hour. The study was approved by UCL’s research ethics committee (ref: 21815/001).

**Table 2:** Outline of each of the measures collected during the pre-intervention and post-intervention research assessments, their method of assessment, and their reliability at pre-assessment

Domain	Variable	Method of Assessment	Reliability Index at Pre-Assessment (Cronbach’s α)
Demographic information			
	Age	Questionnaire	Not applicable
	Ethnicity	Questionnaire	Not applicable
	Sex at birth	Questionnaire	Not applicable
	Gender	Questionnaire	Not applicable
	Sexual Orientation	Questionnaire	Not applicable
	Pubertal Status	Questionnaire	Not applicable
General mental health and wellbeing			
	Strengths and Difficulties Questionnaire [24]	Questionnaire	0.52
	Me and my feelings [13]	Questionnaire	0.70
	Warwick and Edinburgh Mental Wellbeing Scale (WEMWBS; [61]	Questionnaire	0.89
Depression			
	Patient Health Questionnaire-8 [32]	Questionnaire	0.72
Anxiety			
	Generalised Anxiety Disorder Assessment-7 [59]	Questionnaire	0.84
Working memory			
	Backwards Digit Span [51]	Task	Not applicable
Substance misuse			
	Alcohol Use Disorders Identification Tests (AUDIT; [6]	Questionnaire	Not applicable in current sample
	Drug Use Disorders Identification Test (DUDIT; [67]	Questionnaire	Not applicable in current sample
Emotion perception			
	Interpretation Bias Task [47]	Task	Not applicable
	Emotion Intensity Morphing Task [7]	Task	Not applicable
Attributional styles			
	Attributional styles questionnaire [48]	Questionnaire	0.54 (positive subscale), 0.61 (negative subscale)
Positive and negative emotion regulation			
	Emotion reappraisal task—with images	Task	Not applicable
	Emotional reappraisal task—with scenarios	Task	Not applicable
	The emotion regulation Questionnaire for Children and Adolescents [27]	Questionnaire	0.78
Interoception			
	Phase adjustment task [49]	Task	Not applicable
	Interoceptive Accuracy Scale [44]	Questionnaire	0.92
	Interoceptive Attention Scale [20]	Questionnaire	0.92
Social relationships and interpersonal difficulties			
	Peer social networks	Questionnaire	Not applicable
	Inventory of Parent and Peer Attachment [3]	Questionnaire	0.75 (female caregiver), 0.81 (male caregiver), 0.82 (peers)
	Bullying: Multidimensional Peer Victimization Scale [45]	Questionnaire	0.85
	UCLA Loneliness Scale [53]	Questionnaire	0.80

### Measures

We outline the expected outcome measures as planned for the main trial [65], pending confirmation of the feasibility of delivering the assessment battery to the participants in this study (see Table 2 for a summary of the measures). These measures demonstrated acceptable to excellent reliability in the current sample (Table 2). Importantly, to address research questions related to the acceptability of the intervention, we only examined the task-based measures that were delivered in the group intervention. Therefore, the measures described below were not analysed in this paper.

### Qualitative interviews

Six pupils from each participating school (N = 12) were invited to attend an interview about their experience of the intervention. Interviews were conducted at the pupils’ school by a member of the research team who had not been involved in the delivery of the intervention. Interviews were semi-structured and the interview schedule was designed to explore our aims, specifically: experiences of the integration of communication-based strategies from IPT-AST and cognitive-emotion training apps, their experience of the recruitment measures, and barriers and facilitators the young people experienced to participating in the research assessments and intervention. Further questions were asked regarding participants’ experience of benefits and risks of taking part. A full interview schedule can be found in Appendix I.

### ReSET intervention

The ReSET intervention consisted of eight weekly group sessions that last approximately 90 min each, as well as two one-hour individual sessions before the first group session and at the mid-point of the intervention. The intervention begins with a one-to-one session between a young person and one of the group facilitators. The aim of this session is to discuss (i) the aims of the intervention, (ii) the young person’s current relationships as a basis for identifying interpersonal goals for taking part, and (iii) any questions about the group sessions or potential barriers to attending the sessions. In the initial phase of the intervention (sessions 1–4), young people are introduced to the core components of the group including psychoeducational content that highlights the connections between social relationships, emotional processing, and wellbeing. During these early sessions participants are introduced to a curated combination of cognitive-emotional training tasks and communication strategies that could be used to improve interpersonal relationships. For example, participants are asked to discuss how biases in perceiving an individual as friendly or hostile might lead to different reactions, consider timing when approaching someone based on their perceived mood (an interpersonal strategy referred to as ‘Aim for Good Timing’), and role-play how these different scenarios might progress. This interpersonal and communication content is aimed to complement the application of the emotion perception training delivered as part of the cognitive-emotion training (see Interpretation Bias Training, below). Each of the three cognitive-emotion training tasks has associated interpersonal and communication components to help translate the app-based format to an applied scenario (see [37]).

Mid-way through the intervention (between weeks four and five), participants completed a second individual session with the facilitator which assessed pupils’ experience and progress in the group sessions. As in IPT-AST, parents and carers were invited to attend this session to support with use of the strategies at home, though uptake from parents and carers was limited. The middle and closing sessions (sessions 5–8) are designed to help young people actively apply the strategies they had learned to situations relevant to their lives. This is achieved through role-plays, reflections on interpersonal interactions, and homework. For example, a participant would detail an interpersonal conflict (such as an argument with a parent) and the group would discuss ways the strategies could be used to reach a resolution with the individual involved in the scenario. The goal of these later sessions is to prepare young people to use these strategies independently after the conclusion of the group. In each session, participants rated their progress on the Child Outcome Rating Scale (CORS; [8]) as a measure of their therapeutic progress. The CORS measures four domains: The individual’s wellbeing, interactions within their family, their progress in school, and an overall assessment of their wellbeing. In addition, pupils completed the Group Session Rating Scale (GSRS; [14]), which aimed to gather feedback on participants’ experience of the group on a weekly basis. Both the CORS and GSRS could be used to prompt individual check-ins if either measure highlighted areas of concern (see [65]) for further information).

*Interpretation bias training: *The Interpretation bias training task (IBT; [47]) is a computerized training task used to assess and train participants’ biases in perceiving ambiguous facial expressions as friendly or hostile. Participants were required to make a forced choice judgement to indicate if they perceived the face presented as a “happy” or “angry” emotion. Participants first complete 45 trials without feedback, which was used to calculate participants’ bias, or “balance point”, in emotion perception [26, 52]. The balance point is calculated as the total number of faces identified as happy, divided by three.

**Fig. 1:**
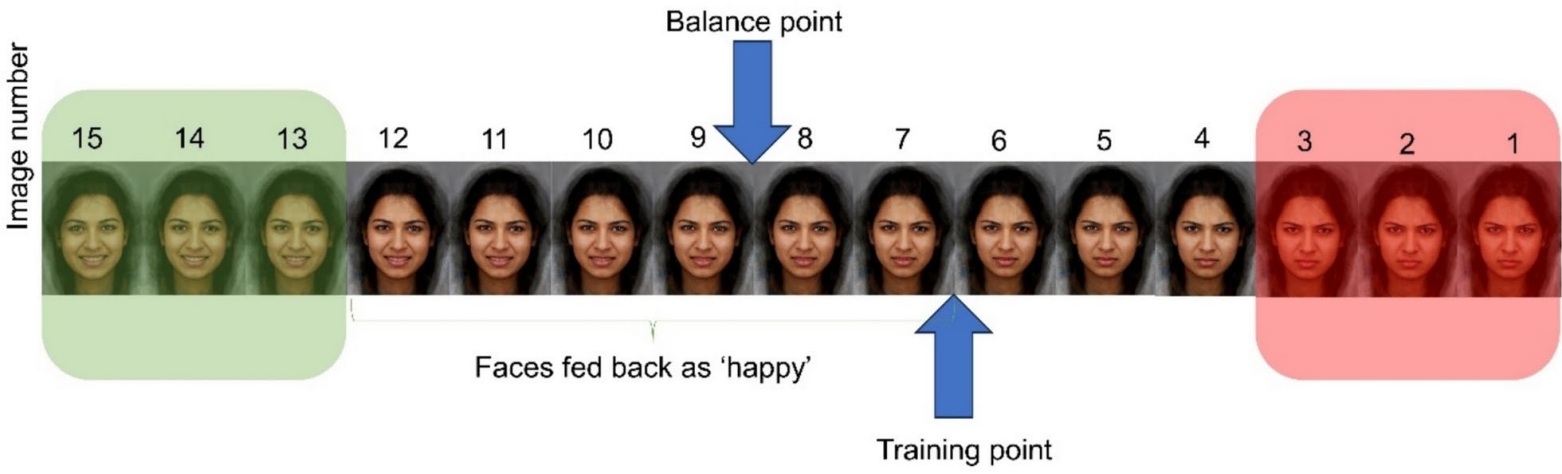
Image depicting the goal of interpretation bias training. The balance point is the point at which the participant exhibits a bias to rate faces as angry. Once this balance point has been calculated, the training component of the task introduces feedback two morphs above the participant’s balance point, indicating the faces at this training point are happy rather than angry. The red shaded images are those which are always fed back as ‘angry’ and green shaded images are those always fed back as ‘happy’, regardless of participants’ balance point

The Interpretation Bias Training procedures used in this study are described in detail in Viding et al. [65]. In the training task, feedback is presented on each trial based on the participant’s balance point. Feedback was calibrated to two points above the participant’s balance point, which was calculated in each training session (see Fig.1). Here, feedback and a visual cue indicating whether participants were correct or incorrect was provided after each response. The feedback was designed to shift participants’ individual bias towards perceiving a greater proportion of stimuli as exhibiting positive emotions, but feedback on the three most unambiguous stimuli would always be congruent with the actual emotion. That is, participants would always receive feedback that the three most angry faces were displaying anger and three most happy faces were displaying happiness. The emotion perception training was completed in each of the group sessions, with the ethnicity and gender of the stimuli differing in each training session.

*Emotion regulation training:*We used a well-established training task for affective cognitive control [23] which was modified for adolescents [1]. The task trains adolescents to employ adaptive emotion regulation strategies, specifically reappraisal and distancing, in negative situations. Participants received developmentally appropriate instructions on the emotion regulation strategies of reappraisal and distancing [25]. Each training block begins with the instruction on which emotion regulation strategy to use, followed by a vignette describing a negative social interaction (e.g., “You have an argument with your family”). Participants were required to imagine the situation and practice the emotion regulation strategy, after which point they are provided with a Self Assessment Manikin rating scale [5] and asked to rate their affect. The emotion regulation training was delivered in six blocks each with six trials. Each block varied by whether participants were instructed to practice reappraising the scenario or distancing themselves from the scenario. The emotion regulation training procedures used in this study are described in detail in Viding et al. [65].

**Fig. 2:**
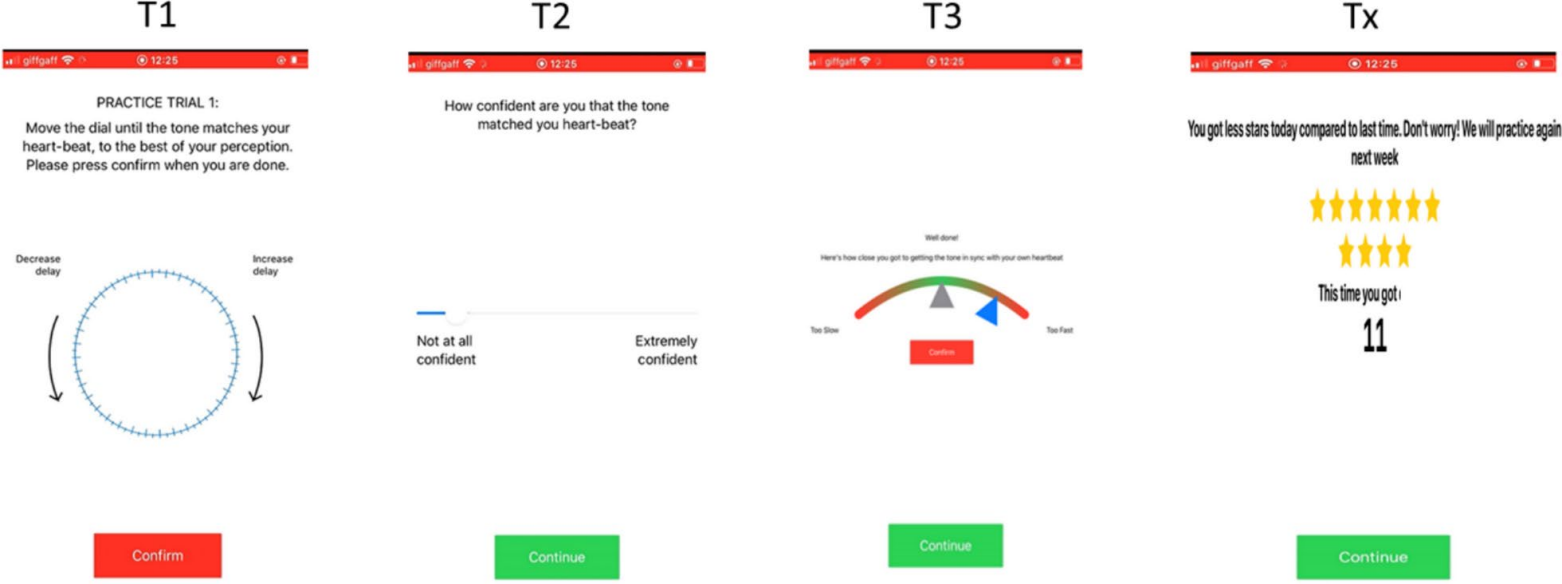
Schematic outline of the Phase Adjustment Training Task. At T1, the participant adjusts the dial until the delay between the beats matches that of their own heart rate. At T2, the participant rates how confident they felt with their score, from not at all confident to extremely confident. T3 is the novel component added in our intervention, where the participant receives feedback based on their accuracy. Marks to the left of the centre indicate the participant was too slow, whereas marks to the right indicate the participant is too fast. The green numbering refers to the number of points collected on the task. Finally, Tx is the final score participant received in the session

*Interoception training:*To train interoception, we developed a novel adaption of the Phase Adjustment Task [49]. In this task, participants were presented with a tone that is out of synchronicity with their heart rate and must adjust a dial to match the tone to their perceived heart rate. However, participants were required to complete a short burst of exercise to raise their heart rate before completing the task, which was designed to draw attention to their heart rate based on an existing protocol [50]. In addition to the exercise designed to increase their heart rate, we also included trial-by-trial feedback demonstrating how close participants’ attempt was to their actual heartbeat. This feedback was visualized as a slider, ranging from ‘too fast’ to ‘too slow’, with the centre denoting that participants were close to their actual heart rate. If participants’ attempt was towards the centre of the slider, they would receive stars (ranging from 1 to 3) to reward their performance. At the end of training session, they would receive feedback about the total number of stars they had collected in that training session (see Fig.2). Participants completed eight trials of the training task, which took approximately eight minutes in total.

### Data analysis

To assess the first aim of the study (i.e., the feasibility of programme delivery and whether it could be delivered as a cohesive intervention), we used qualitative data to assess participants’ understanding of the links between the interpersonal strategies and the cognitive-emotional training tasks. Acceptability of the intervention was examined using qualitative data to identify any positive or negative experiences of the intervention, as well as quantitatively using attendance data to examine whether participants opted not to complete the intervention, which would indicate poor acceptability. An additional consideration of the programme feasibility was whether the group-based delivery of the intervention could reduce the performance of the cognitive-emotional training tasks, which have typically been delivered on a 1:1 basis. These previous studies have demonstrated that participants’ performance have improved in line with the presumed mechanism of change on training tasks. Specifically, previous work has demonstrated that participants’ balance point changes towards viewing a greater proportion of faces as friendly, rather than hostile, on the interpretation bias task [47], participants ability to regulate their affect improves through emotion regulation training [74], and participants’ interoceptive accuracy increases with interoception training [50]. To provide indication if the training tasks worked as intended in a group format, we drew on two quantitative data sources:

(i) Training data from participants allocated to the intervention group. Here, we examine in the trajectories of participants’ performance on the cognitive-emotional training tasks, using graphical displays of data
(ii) Pre- and post-intervention data compared between intervention and control participants. For these data, we use descriptive statistics and graphical displays to examine change in the intervention participants’ data from pre- to post-intervention, and whether this differs for participants in the control arm of the feasibility study.

The second aim of this study was to evaluate the robustness of our identification and recruitment procedures as delivered in a school setting. To evaluate this aim we examined the number of pupils that progressed from the initial screening to completing the follow-up research assessments. We compared these figures against several benchmarks: (i) whether the threshold used identified approximately 25% of the year group scoring highly on measures of psychopathology, as this was the percentage used to establish the threshold [62], (ii) whether at least 50% of eligible pupils consented to take part in the study and (iii) whether at least 80% of pupils were retained from baseline assessments to follow-up assessments. Benchmarks for (ii) and (iii) were drawn from the sample size calculations detailed in Viding et al. [65]. For each stage of the recruitment procedure, qualitative data were used to complement the quantitative measures to gain an understanding of participants’ perspectives on the recruitment procedures.

Finally, our third aim was to evaluate the feasibility of delivering the required trial procedures for evaluating ReSET and in particular the completeness of pre- and post-outcome assessments on both arms of the study in a school-based setting. To examine this aim, we report descriptive statistics on the completeness of the questionnaire and task data (see Table 2) to assess whether the data could be collected with minimal data loss. However, we did not conduct any analyses on the assessment measures.

## Results

### Aim 1: examining the acceptability of the ReSET programme

The qualitative data suggested that participants were able to understand the link between the group-based interpersonal content and the cognitive-emotional training tasks, suggesting they experienced the intervention as one cohesive programme. For example, when asked about whether they found the cognitive-training tasks helpful, one participant reflected on applying the Interpretation Bias Training to their real-world choices, and subsequently combining it with the interpersonal strategy ‘Aim for Good Timing’ to evaluate when to engage in conversation: “*When a person feels angry, upset, a negative emotion—I can tell by the looks of it, I don’t even have to ask… If they’re happy, chatty, smiling, laughing, I will go up and talk to them, not back off*.” Similarly, another participant reported using the reinterpretation emotion regulation strategy to consider alternative reasons for a scenario and combined this with the interpersonal strategy of ‘Putting themselves in Somebody Else’s Shoes’*.* In answer to a question about what strategies they found helpful, one participant stated: *“Try and look at it from a different perspective… because it kind of goes under putting yourself in other’s shoes. Because it does sort of correlate*”.

As further evidence of the acceptability of the hybrid intervention, reflections from participants were overwhelmingly positive about their experiences of the programme. Participants stated that the groups were “*really fun*” and importantly, that their experience of the group positively impacted their mental health, as they felt “*lighter*” after having completed the groups. This qualitative data was further corroborated by quantitative data demonstrating high attendance in the intervention groups (133 out of 160 total sessions attended across both groups). Attesting to the positive experience of the group, one participant reflected:

“*When I first started I was, I wouldn’t say depressed because I was a worrying child, but in terms of my life being exciting or interesting, it wasn’t really interesting—football, eat, sleep, repeat—and it was kind of like that, so it wasn’t really interesting. But now I feel more happier, like a life to look forward to… the group helped me feel like that.*”

In addition, participants also reported developing new friendships during the intervention sessions or strengthening existing relationships with their peers, which was an additional benefit of taking part. For example, one participant reflected: “*I found the groups helpful and I found the groups really nice, and I like the fact that I made pretty much new friends*.”

**Fig. 3:**
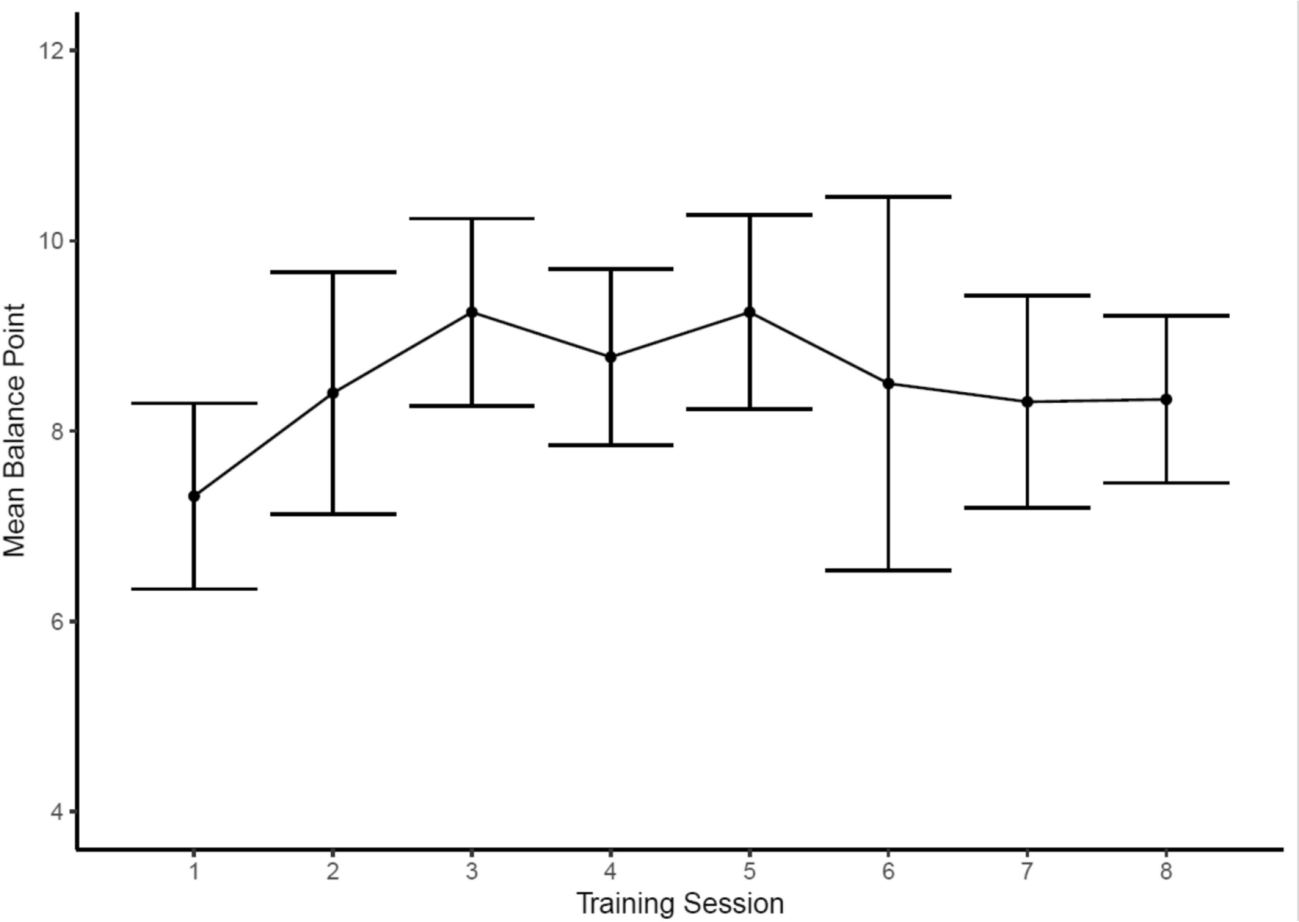
Emotion perception training data demonstrating a shift in participants’ (N = 20) mean balance point (i.e., the point at which they rate faces happy, compared to angry). Note, higher scores indicate participants are rating a greater proportion of faces as happy. Bars around each point indicate 95% confidence intervals

**Fig. 4:**
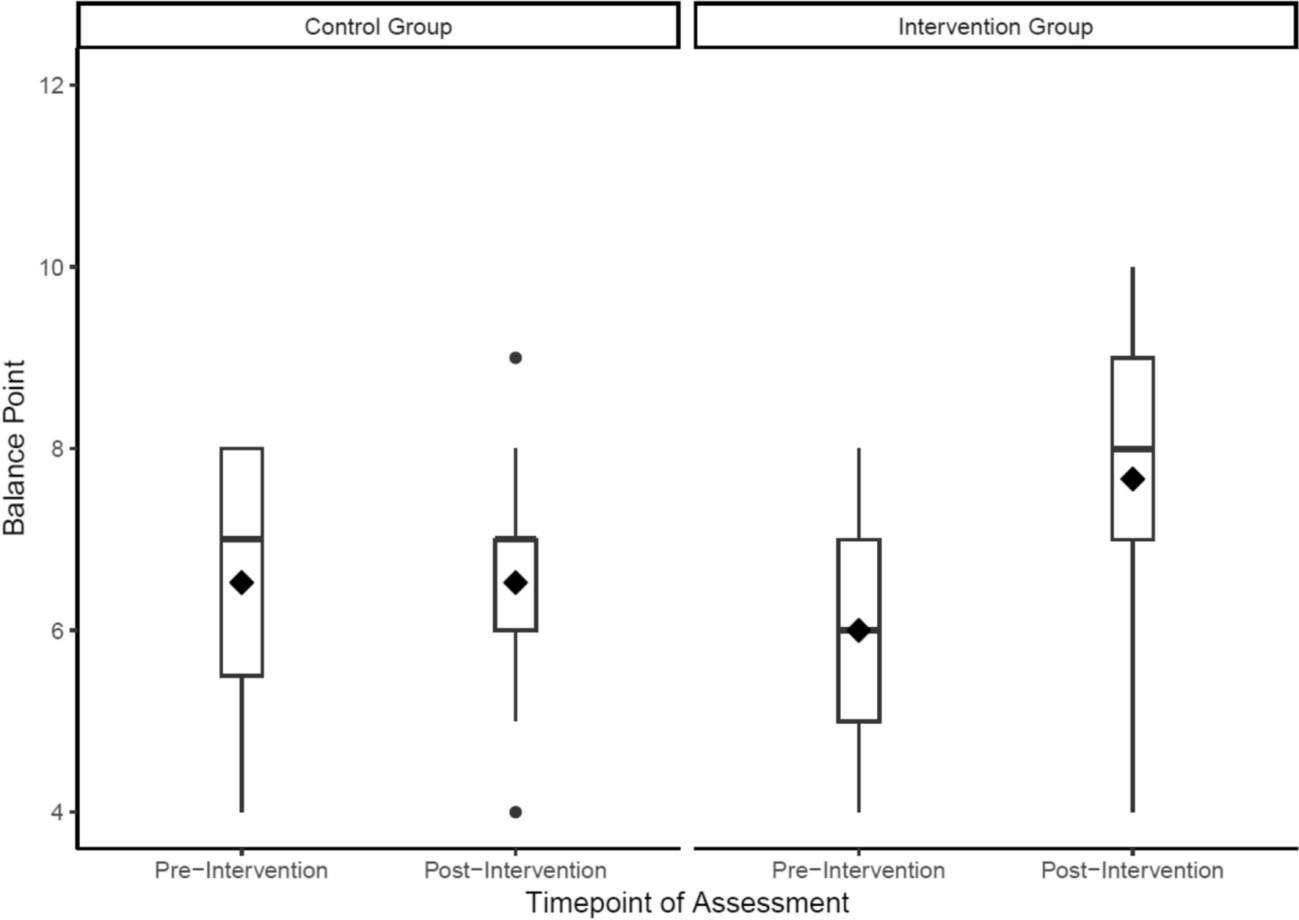
Emotion perception assessment data demonstrating the intervention group (N = 20) had a higher mean balance point after the intervention, compared to before the intervention. In contrast, the control group (N = 20) exhibit no change in their mean balance point. Diamonds indicate the mean for each group at each timepoint, and circles indicate outliers

To assess adherence to the cognitive-emotional training protocols, we examined trajectories for each of the three emotion processing domains trained in the intervention: Emotion perception, emotion regulation and interoception. These data suggested that the emotion perception training could be successfully delivered in a group-based setting. Consistent with previous research [47], participants’ balance point increased over the course of the training sessions, indicating a higher proportion of faces were identified as happy rather than angry, though we noted a decline from sessions five onwards. The successful operation of the emotion perception training was further supported by the assessment task data, where we observed an increase in intervention participants’ mean balance point, whereas this change was not observed in control participants (see Figs. 3 and 4). Specifically, there was an increase in the intervention group’s mean balance point from the baseline assessment (M = 5.70, SD = 1.45) to the follow-up assessment (M = 7.47, SD = 2.03). In contrast, there was no notable change in the control group’s mean balance point from the baseline assessment (M = 6.52, SD = 1.50) to the follow-up assessment (M = 6.39, SD = 1.33). Qualitative data indicated positive experiences of this training protocol, as well as participants’ ability to apply it to their everyday lives:

*“I liked was the faces where you read the faces of the person, I liked that because it kind of showed me how to read different emotions. Even sometimes they could be looking like that and different level of that kind of thing.”*

**Fig. 5:**
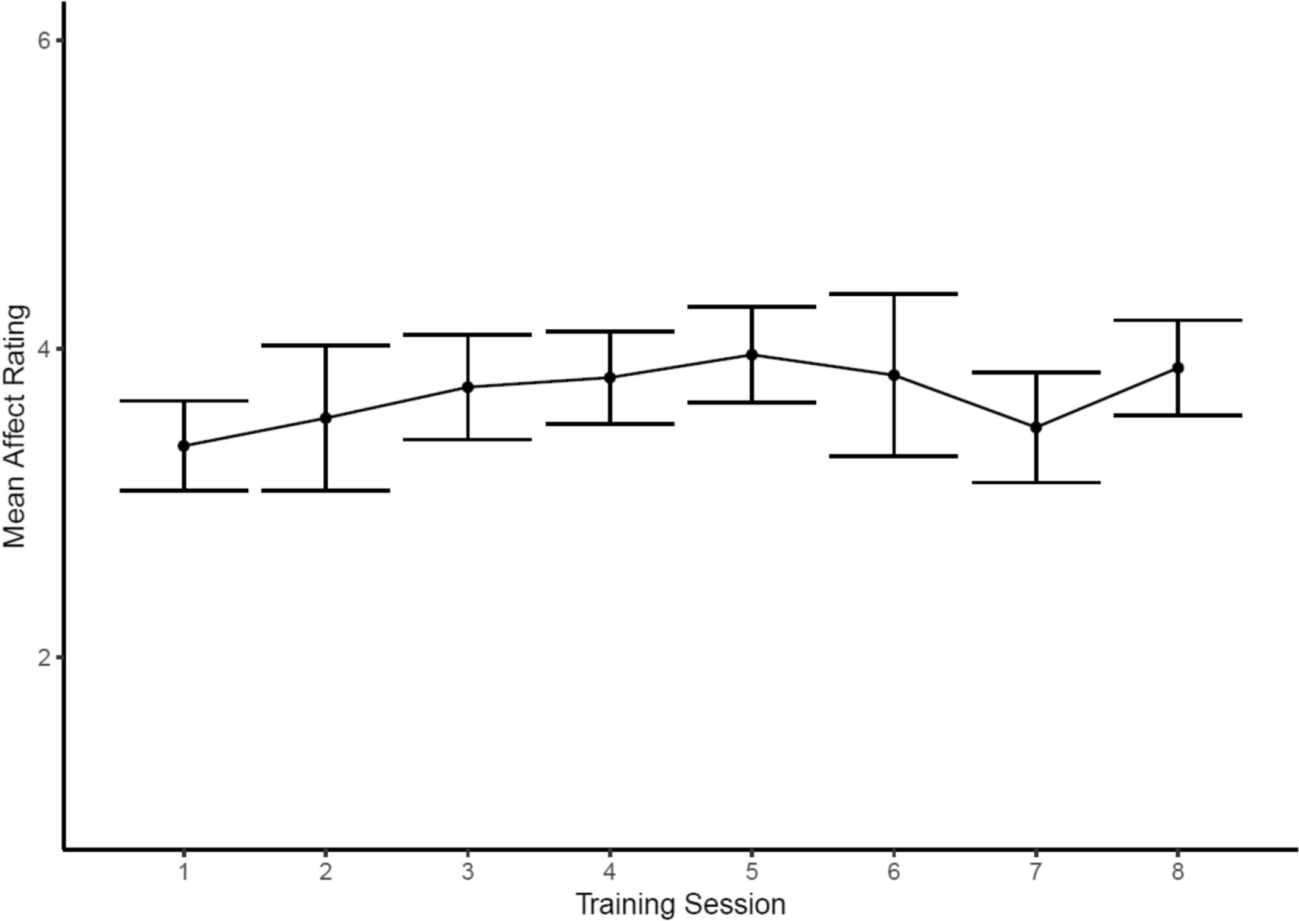
Emotion regulation training data from the intervention group (N = 20) showing an increase in mean affect, rated after practicing an emotion regulation strategy (either reinterpretation or distancing), over the course of the intervention. Bars around each point indicate 95% confidence intervals

**Fig. 6:**
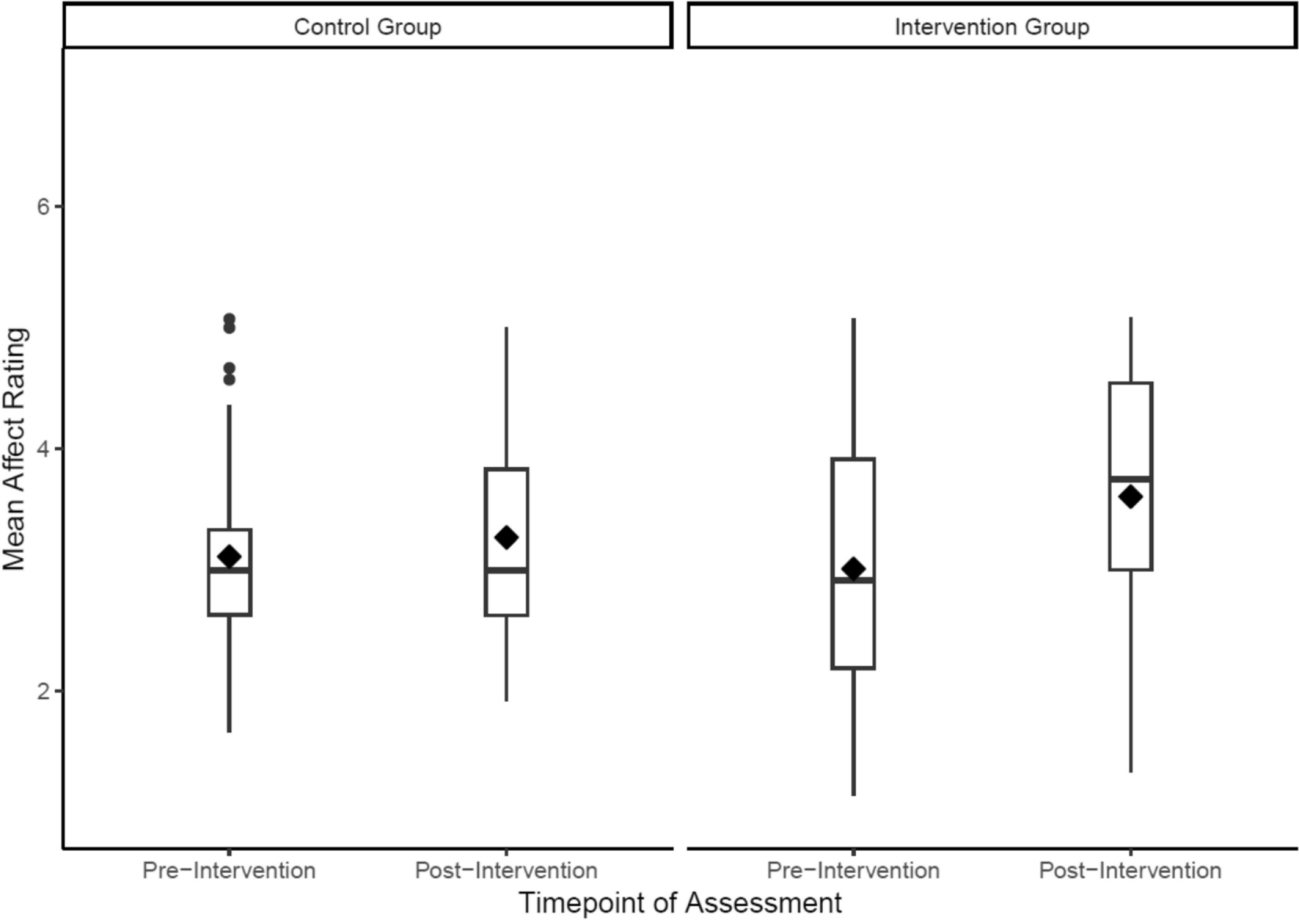
Assessment data for the emotion regulation task. These data depict trials where participants are instructed to reduce their negative affect and suggest that intervention participants (N = 20) have an improved ability, relative to the control group (n = 20), to regulate their emotions after the intervention. Diamonds indicate the mean affect rating for each group at each timepoint and circles indicate outliers

Similar to the emotion perception training, we found improvements in self-reported affect following the emotion regulation training when participants practiced the two emotion regulation strategies taught in the intervention, indicating that the training was working as planned. Participants reported feeling less negative affect in response to negative scenarios the further they progressed in the intervention (see Fig. 5). These data offer encouraging evidence for the efficacy of the emotion regulation training when used in our group setting intervention. In addition to these training data, we also observed improvements in the assessment data between the intervention and control groups, in which participants were instructed to reduce their negative affect in response to a presented scenario. There was a modest increase in the intervention group’s mean affect ratings (indicating an improved ability to regulate emotions) from the baseline assessment (M = 2.89, SD = 0.88) to the follow-up assessment (M = 3.33, SD = 1.07). There was slightly less change in the control group’s mean affect ratings from the baseline assessment (M = 3.03, SD = 0.68) to the follow-up assessment (M = 3.25, SD = 0.85; Fig. 6). Indeed, qualitative data indicated participants were able to utilize the strategies trained in this task: “*one of the tasks was to reinterpret something, like a situation to make it more positive… I've been using that a bit*.”

**Fig. 7:**
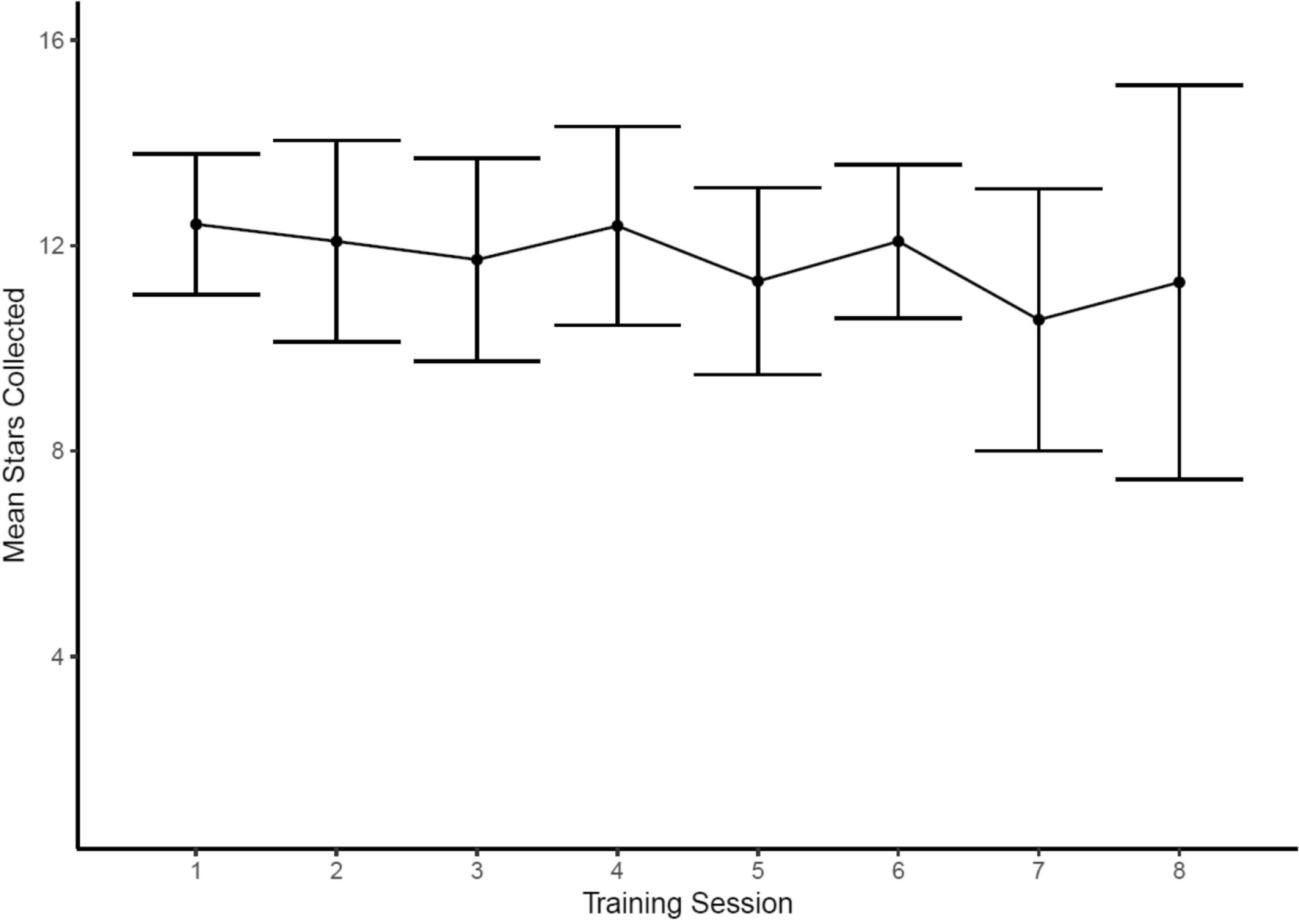
Plot demonstrating that intervention participants (N = 20) collected on average a similar number or fewer stars (i.e., were similarly or less accurate at matching their heartbeat to the tone) as they progressed through the intervention. Bars around each point are the 95% confidence intervals. Fewer participants completed training session 8, contributing to the wider confidence intervals in this session

The results for the interoception training task indicated that the training task was not working as intended in a group format, as participants’ interoceptive ability did not improve across the training sessions and instead appeared to decline over the course of the intervention (see Fig. 7). Further, during the assessment phase of the Phase Adjustment Task, individuals are assigned a status based on whether they provided consistent ratings, in which case they were classified as interoceptive, or non-interoceptive if their rating were inconsistent (see [49] for further information). Examining the assessment data, the proportion of pupils classified as interoceptive declined from baseline assessments to the follow-up assessment (see Table3).

**Table 3:** Pre-assessment and post-assessment classification of interoceptive status of study participants

	Pre-assessment (N = 41)	Post-assessment (N = 37)
Interoceptive participants (total)	21 (51%)	10 (27%)
Interoceptive participants (intervention group)	11 (52%)	3 (14%)
Interoceptive participants (non-intervention group)	10 (50%)	7 (35%)

These data demonstrate that the proportion of participants classified as ‘interoceptive’ declined from the pre-assessment to the post-assessment in both intervention and control groups. However, this decline was particularly pronounced in the intervention group. These findings could suggest that the intervention was leading participants to become poorer at identifying their internal bodily signals (i.e., causing iatrogenic harm). However, our qualitative feedback suggested that the decline in participants classified as interoceptive was due to disengagement with the task, rather than their interoceptive ability becoming poorer: “*I didn’t really understand the heartbeat one, like what you had to do. I understand what it was for, but I don’t understand what you actually had to do.*” Notably, this excerpt highlights a theme that was prevalent across study participants where they acknowledged the importance of interoception as a skill but struggled with the design of the training task. Supporting this interpretation, comparing the completion times between the pre-assessment (M = 17.51, SD = 11.15) and post-assessment (M = 10.89, SD = 3.26), there was a marked decline in the completion time at the post-assessment timepoint, which likely indicates that the participants were rushing through the task without engaging with it and did not adhere to the protocol, given their reported dislike of the task (see Table 4).

**Table 4:** Completion time (in minutes) in the ReSET study, split by interoceptive status

	Interoceptive	Non-interoceptive	Unclassified
ReSET feasibility (pre-assessment)	M = 20.5, SD = 14.8, N = 21	M = 14.6, SD = 3.7, N = 8	M = 14.2, SD = 3.5, N = 12
ReSET feasibility (post-assessment)	M = 11.0, SD = 2.1, N = 10	M = 12.1, SD = 5.4, N = 8	M = 10.4, SD = 2.8, N = 19
Plans et al. [49]	M = 19.8, SD = 12.6, N = 8	M = 17.7, SD = 10.7, N = 14	NA

For comparison, we include data from published study examining task performance in adults [49]

### Aim 2: evaluating the feasibility of the recruitment procedure

**Fig. 8:**
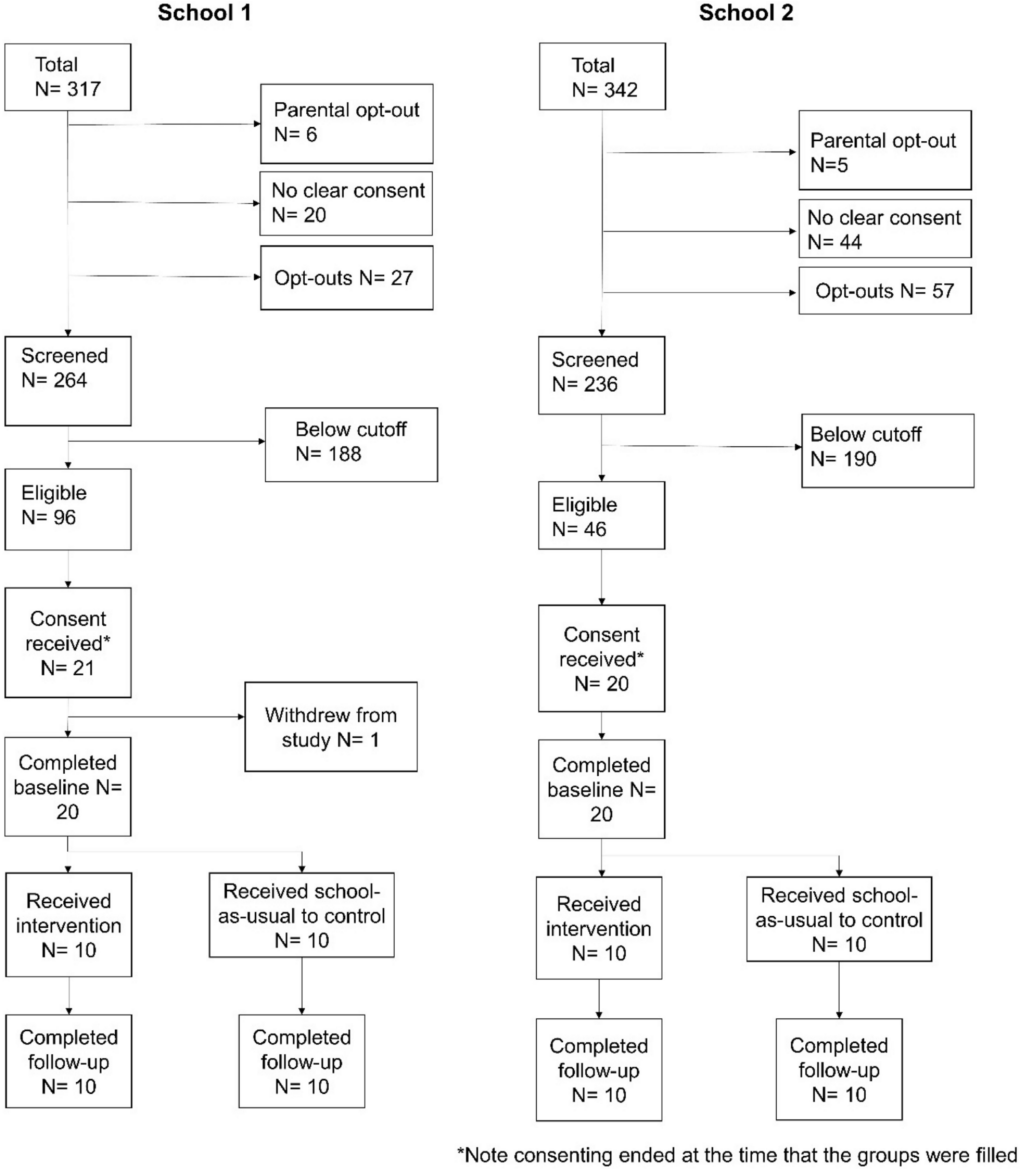
CONSORT Diagram demonstrating the recruitment and retention of participants throughout the feasibility study

The second aim of the study was to assess the feasibility of the recruitment protocols, including the screening measure, consent procedures, and retention. In School 1 there was evidence that our consent procedure for the screening measure was successful, as 85% of the year group consented to complete the screening questionnaire (see CONSORT Diagram; Fig. 8). In School 1, 36.4% of screened pupils were above the SDQ cut-off. The cut-off we applied for the current study was based on the top quartile drawn from a nationally representative sample [62], meaning the rates of participants in School 1 scoring above 15 was above this nationally representative sample. In School 2 these figures differed insofar as the consent rates for the screening measure were much lower at 70.0%. Further, of the pupils that completed the screening questionnaire, the number eligible to take part in the study was 19.5%.

Regarding their experiences of completing the screening questionnaire, participants did reflect that they were concerned about the confidentiality of their answers and their responses being viewed by their peers, but were generally happy to complete the measure. For example, one participant reflected: *“I don't think I minded it, it wasn’t anything super stressful… but I also didn’t want people looking at my answers.”*. However, when asked about their reflections on completing the screening measure, they stated it could be improved with “*more explanation on why we’re doing it*.” Notably, this participant was recruited from School 2 and may suggest the lower consent rates from this site (relative to School 1) were related to participants’ lack of understanding regarding the purpose of the questionnaire.

Of pupils eligible to take part in the study, we received consent for 21.9% of pupils at School 1 and 43.5% of pupils at School 2 (32.7% aggregated across both schools). The analysis plan for the main trial [65] assumed a consent rate of 50% of all pupils identified as being eligible from the screening measure, meaning these rates were slightly lower than anticipated for the main trial. However, it is important to note that we did not seek consent from additional pupils after reaching the 20 pupils required from each school to run the intervention and control groups. Further, the retention rates from baseline to follow-up assessments was higher than the 80% projected for the main trial [65], as 98% of participants (40 out of 41) recruited to the intervention and control groups completed the follow-up assessments. The single pupil loss to attrition was due to self-withdrawal during the baseline assessments.

An additional measure of the feasibility of delivering the programme is how group attendance may be affected due to illness, pupils opting to attend their usual classes, or clashes with the school schedule. To examine this possibility, we calculated attendance rates within the intervention groups. In School 1, there was 77.5% attendance across all eight sessions and in School 2 this figure was 88.8% (83.1% aggregated across both schools). The most common reasons pupils reported absences were due to illness (66.7% of all absences), though for a small number of sessions there were clashes with other school activities (e.g., exams or external trips) that meant pupils were unable to attend the sessions (22.2% of all absences). A small proportion of absences (11.1%) were due to participants opting to attend their usual classes rather than the intervention sessions. Despite the small number of absences across the intervention, these data suggest both the intervention could be successfully delivered in the school setting.

### Aim 3: feasibility of the research measures

The third aim of this study was to examine the feasibility of delivering the assessment battery in a school-based setting while avoiding data loss. Of the questionnaire data, 3.6% was recorded as missing. For the AUDIT and DUDIT, participants were not asked to complete questions 2–9 if they reported no alcohol or drug use in question 1 and therefore these questions were not recorded as missing if participants had not reported alcohol or drug use in response to question 1. We recorded 11.5% of AUDIT data as missing and 12.8% of DUDIT data as missing. With regards to the task-based measures, missing data were defined as participants not having recorded an attempt at the task. Across the 6 behavioural tasks (interpretation bias, the two emotion regulation tasks, the backwards digit span, the emotional intensity morphing task, and the Phase Adjustment Task), we recorded 2.5% of missing data across the two assessment timepoints in both intervention and control groups. Indeed, participants reported positive experiences of completing these measures, perhaps explaining the high completion rate: “*It was actually kind of helpful and made me like how I was feeling then and feeling now and cool to see the difference*.”

## Discussion

The aim of this study was to examine the feasibility of a novel, transdiagnostic, indicated prevention intervention for adolescent mental health: The ReSET programme. Quantitative and qualitative data from this study supported the view that the intervention was acceptable to participants, with those taking part in the programme reporting positive experiences of the intervention group. Participants reported using a combination of the strategies adapted from IPT-AST and cognitive neuroscience, suggesting the intervention had been experienced as a cohesive programme. We found good evidence for acceptability of the emotion perception and emotion regulation training tasks. In contrast, participants did not understand the interoception training protocol and the task did not appear acceptable to study participants. Further, data from this study indicated that the delivery of the intervention was feasible within a school setting; we successfully recruited and retained participants in both intervention and control arms of the study, broadly in line with sample size calculations for the main trial [65]. In addition, attendance to the ReSET programme was generally high and were able to deliver the research measures to intervention and control participants with little data missingness. Altogether, these findings provide evidence of the feasibility of the intervention and motivate further study into the mental health benefits of the programme through a full-scale RCT.

### Integrating the cognitive emotion training tasks into a group-based psychosocial intervention

Data from the feasibility study indicate the ReSET intervention, integrating communication-based strategies from IPT-AST and cognitive-emotional training tasks, was experienced as a cohesive intervention by participants. Notably, several participants reported combining communication and app-based strategies in real-world settings, suggesting that there is a potential advantage in combining of the two intervention approaches to a single intervention protocol. Indeed, participants’ reflections on the application of the strategies suggests that they were not experienced as distinct components, overcoming the challenge of finding a common language across fields as reported elsewhere [37]. The benefit of integrating interventions is highlighted by previous work that demonstrated participants who were matched to an intervention that aligned with their symptom profile (i.e., either interpersonal or cognitive-emotional) benefitted more from these interventions compared to participants who completed interventions that were not matched to their symptoms [68]. By providing a hybrid intervention that targets both social and cognitive-emotional mechanisms implicated in mental health problems, the ReSET programme has the potential to be an effective, transdiagnostic intervention for a broad range of adolescents at risk of psychopathology.

With regards to the cognitive-emotional training tasks, the data suggest that participants’ bias to perceive faces as happy or angry changed during the intervention, with the assessment data suggesting this ability was markedly changed compared to the non-intervention group. Similarly, participants’ ability to regulate their negative emotions seems to have improved during the intervention, with some evidence suggesting improvements only in the intervention as compared with non-intervention group. These findings support the efficacy of the cognitive-training tasks and their novel integration into a group-based intervention. However, one observation from the emotion perception training task was that participants’ balance point declined from session six onwards (i.e., participants began to view a greater proportion of faces as hostile from session six onwards). This observation could reflect three phenomena: First, as participants reach a balance point that is optimal (i.e., rating the majority of faces as happy with the exception of the three unambiguously angry faces; see Fig. 1), this may increase the salience of feedback indicating faces are angry due to this feedback being received relatively less frequently. This rarer feedback may shift participants’ balance point to view more faces as hostile rather than friendly, contrary to the aims of the training. A second possible explanation for the decline in balance point from session 6 onwards is that participants are trained to the point of credibility, such that participants no longer consider the feedback valid. Indeed, previous research has typically administered five training sessions to avoid this issue [52], whereas we included eight training sessions in the intervention. A final possible explanation for these data is that it could indicate disengagement from the task. In response to these observations, we opted to reduce the number of emotion perception training sessions from eight to six [65].

The data did not appear to support the use of the interoception training in this context, as the quantitative data suggested that participants’ performance on the interoception training task became poorer during the course of the intervention. However, examining data about participants’ response times between pre- and post-assessments suggests that the decline in performance was likely due to participants disengaging from the task. Indeed, this was corroborated by qualitative data with participants reporting a dislike of the task and did not report benefiting from completing the training. These findings suggest that, unlike the emotion perception and emotion regulation training, the interoception training protocol did not work in a group setting.

In response to the decline in interoception performance in the feasibility study, we opted to remove this training task from the final intervention [65]. Discussions within the study team highlighted that attempts to motivate participants to complete the interoception training task were potentially disrupting the rapport-building between facilitators and intervention participants [37]. Moreover, the time taken to complete this activity detracted from other beneficial activities that could be completed during the intervention sessions. When considering reasons that interoception training was not successful in the current intervention, possible reasons include that participants could not focus on the activity in group-based settings. The ability to attend to internal physiological sensations is challenging even when completed alone by adults [49, 50]. Therefore, it is possible that external distractions in group settings made this activity additionally challenging, which may produce a negative feedback loop between performance and distraction, leading to disengagement from the task [73]. Moreover, participants did not appear to understand the task, suggesting future work could improve the clarity of the instructions potentially by including an element of biofeedback. However, we retained discussion activities about the importance of attending to internal physiological signals in the main intervention [30], as our qualitative data indicated that participants understood the importance of this ability, but specifically struggled with the task. To examine whether interoceptive ability can be successfully trained using task-based training, future research should consider trialing interoception training with adolescents in individual sessions, rather than in a group-based setting.

### Feasibility of delivering ReSET as a school-based intervention

The second aim of this study was to examine the feasibility of delivering the recruitment and research methods in a school setting. One observation from the feasibility study was the difference in the number of unclear consents or explicit opt outs between Schools 1 and 2, with a higher proportion of pupils in School 2 deciding not to complete the screening questionnaire. We suggest that these differences in response rates could be due to the differences in how relationships were built between these sites. School 1 was involved in the study from the project inception and several rounds of knowledge dissemination had occurred in this school about the study, prior to the screening questionnaire being delivered. In contrast, School 2 was recruited with shorter notice, meaning that fewer relationship-building and dissemination activities were conducted. Indeed, this interpretation is corroborated by our qualitative data, where participants in School 2, but not in School 1, reflected that they would have benefitted from greater information about the study prior to completing the screening questionnaire, which might improve consent rates. These findings highlight the need for a robust recruitment strategy that provides participants with information about the study well in advance prior to conducting the screening and assessment measures [39].

Delivering school-based mental health interventions is challenging in the context of the multiple competing demands on both pupils and school practitioners [39]. It was therefore vital to establish that the intervention and research assessments could be successfully delivered in this setting prior to launching a full-scale trial. Data from this study suggest that the intervention could be successfully delivered in schools as we observed limited absences from the intervention sessions. Where absences were reported, only a small proportion of these were due to pupils wishing to attend their regular classes rather than the intervention sessions. This highlights the importance of monitoring individual pupil motivation and taking steps to address potential motivational issues for a small subset of pupils at different points of the intervention. In addition, we recorded only minimal data missingness, indicating it was feasible to deliver an assessment battery during school hours both before and after the intervention.

### Benefits and risks of taking part

Data from the feasibility study generally supported the view that participants had positive experiences from taking part in the intervention, and there were limited risks to their participation. The qualitative feedback from participants was overwhelmingly positive about the benefits of the intervention, including leading to indirect positive outcomes, such as establishing new friendships– a predictor of positive mental health outcomes in adolescence [64]. Moreover, several participants directly attributed improvements to their mental health to taking part in the intervention, suggesting the intervention has potential to improve symptomology in this population. We therefore consider this encouraging evidence for the benefits of taking part, with limited risk to study participants.

However, a limitation of the feasibility trial was that we were unable to trial our randomisation protocol across two sites, as one school had already agreed to participate in the main trial, and we therefore wanted to avoid screening a year group that would be eligible for the study. Participants in the main trial are assigned to the intervention at year-group level, with randomisation occurring across schools as to which year group receives the intervention. This randomization procedure is detailed in Viding et al. [65]; however, we are only able to report on the feasibility of this procedure at one site. However, we note at the one site where this randomization procedure was trialed, no issues were observed. A further limitation is that the two groups were either led by, or run with the support of, a member of the research team. The intention for the main trial is that intervention groups should be led by a trained member of school staff or mental health professionals working in schools (e.g., Education Mental Health Practitioners). Although this decision was made, in part, because the intervention was still under development, it means we are unable to assess the feasibility of delivering the intervention when led entirely by staff external to the research team.

## Conclusions

Given the increasing rates of mental health problems among adolescents [15], it is important to develop accessible, effective and scalable interventions to prevent the onset or worsening of mental health problems. This programme contributes to ongoing efforts to develop school-based interventions and systematically evaluate their efficacy [2, 34]. We have innovated on existing school-based interventions by developing an indicated, preventative, transdiagnostic intervention for adolescents, in contrast to previous interventions that have adopted either universal or diagnostic approaches (e.g., [46]). Should the efficacy of the intervention be demonstrated through a full-scale randomised controlled trial, this intervention has the potential to benefit adolescents by preventing the escalation of mental health problems and the downstream sequalae of negative consequences associated with psychopathology.

In sum, this study was a feasibility evaluation of a novel preventative, indicated, transdiagnostic, school-based intervention. We found promising evidence for the benefits of the intervention from qualitative feedback, with participants reporting benefits arising from the novel combination of the two types of intervention (IPT-AST and cognitive-emotional training). The study also demonstrated acceptability and feasibility of the programme, as the intervention and research assessments were successfully delivered in schools with minimal attrition. Data from the feasibility study was also informative in optimising the intervention and study measures for the full-scale trial, including removing the interoception training from the intervention. Together, these findings highlight the potential for the ReSET intervention to be delivered in schools, with the opportunity to alleviate symptoms of psychopathology in adolescents with heightened risk of experiencing mental health problems.

## Supplementary Information

Supplementary Material 1

## Data Availability

The datasets generated and/or analysed during the current study will be available in the Open Science Framework (OSF) repository: https://osf.io/34jur/?view_only=b3963b4b23f94c3aa463cc92453169c7.

## Abbreviations

AUDIT: Alcohol Use Disorders Identification Tests
CORS: Child Outcome Rating Scale
DUDIT: Drug Use Disorders Identification Test
GAD-7: Generalised Anxiety Disorder Assessment-7
GSRS: Group Session Rating Scale
IBT: Interpretation Bias Training
IPT-AST: Interpersonal PsychotherapyAdolescent Skills Training
M&MF: Me and My Feelings
PAT: Phase Adjustment Task
PHQ-8: Patient Health Questionnaire-8
ReSET: Building Resilience Through Socioemotional Training
SDQ: Strengths and Difficulties Questionnaire
WEMWBS: Warwick and Edinburgh Mental Wellbeing Scale
