# Supplementary material for: A feasibility study of a preventative, transdiagnostic intervention for mental health problems in adolescence: building resilience through socioemotional training (ReSET)
Source: Child Adolesc Psychiatry Ment Health. 2025 Mar 22;19:29. doi: 10.1186/s13034-025-00870-z (PMC11929178; doi:10.1186/s13034-025-00870-z)
Supplement: Supplementary file 1 — Supplementary Material 1 [file 13034_2025_870_MOESM1_ESM.docx]

**Supplementary Materials: A Feasibility Study of a Preventative, Transdiagnostic Intervention for Mental Health Problems in Adolescence: Building Resilience Through Socioemotional Training (ReSET)**

**Supplementary Aims**

In addition to our primary aims, we had several secondary aims in the feasibility study. The first was to identify any potential risks from taking part in the study. Recent large-scale universal school interventions have demonstrated evidence of iatrogenic harms in these studies (Andrews et al., 2023; Kuyken et al., 2022). We therefore measured participants’ mental health and wellbeing before and after the intervention to examine whether the programme was associated with any iatrogenic harms. This process also included procedures to monitor adverse events through taking part in the intervention. Further, we also examined several mechanisms that may mediate the efficacy of the intervention including emotion perception, emotion regulation, interoceptive ability (Viding et al., 2024). Another secondary aim of the feasibility study was to examine whether, relative to the control group, the intervention group demonstrated any descriptive differences in these mediating mechanisms after completing the intervention.

**Supplementary Results**

The secondary aims of the feasibility study were to assess the benefits and risks of the intervention. We conducted descriptive analysis of participants’ CORS scales to identify trends in their wellbeing over the course of the intervention. Further, we report descriptive statistics related to the mediating mechanisms measured pre- and post-intervention. There was evidence that the intervention produced positive outcomes for the pupils who took part in the ReSET intervention. In each session, participants rated their life functioning across several domains using the CORS measure. These data indicated that across the eight sessions, participants’ life functioning generally improved with the exception of the school domain (see Supplementary Figure 1). We note that the feasibility study was conducted at the beginning of the academic year, which may have affected pupils’ ratings of their school experiences during the course of the study.

*
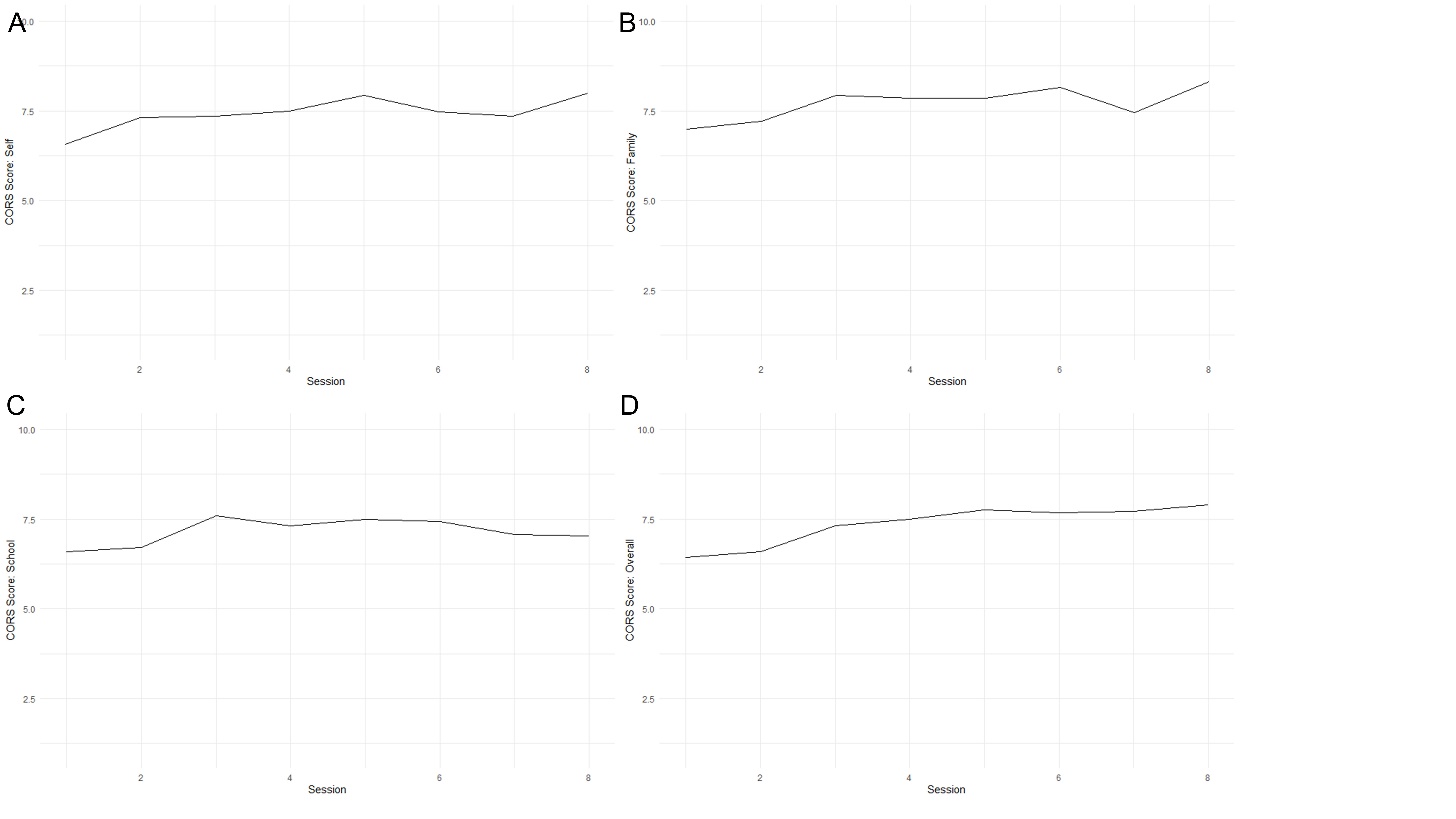
*

*Supplementary Figure 1: Plots demonstrating average scores on each of the four CORS domains for the participants assigned to the ReSET intervention, measuring sarisfaction with self (plot A), family (plot B), school (plot C) and their overall satisfaction (plot D).*

We also provide descriptive summaries of the changes to questionnaire measures recorded at the pre-assessment timepoint and post-assessment timepoint for both intervention and control groups (see Supplementary Table 1). Although these data do not indicate improvement in the intervention relative to the control group, we note that the sample size was too small to draw any conclusions from these descriptive statistics. Importantly, these data do not suggest any iatrogenic harms were associated with participating in the ReSET programme.

*Supplementary Table 1: Means and standard deviation of the questionnaire assessment measures at pre-assessment and post-assessment for the intervention and control groups.*

| **Measure** | **Control Group Baseline Assessment Mean (SD)** | **Control Group Post-Assessment Mean (SD)** | **Intervention Group Baseline Assessment Mean (SD)** | **Intervention Group Post-Assessment Mean (SD)** |
| --- | --- | --- | --- | --- |
| SDQ Total Scores | 26.53 (3.84) | 26.56 (5.44) | 26.68 (5.12) | 27.32 (5.60) |
| Me and My Feelings | 30.82 (4.13) | 29.78 (6.16) | 32.47 (4.45) | 31.16 (6.30) |
| WEMWBS | 39.47 (7.87) | 42.17 (10.60) | 44.26 (10.24) | 41.89 (8.76) |
| ASQ (Negative) | 6.94 (2.60) | 7.47 (2.76) | 5.89 (2.49) | 6.22 (2.18) |
| ASQ (Positive) | 4.17 (2.19) | 4.88 (2.09) | 4.68 (2.19) | 4.94 (2.19) |
| ERQ (reappraisal) | 3.06 (1.26) | 3.00 (0.97) | 3.42 (0.77) | 3.68 (0.82) |
| ERQ (suppress) | 4.24 (0.83) | 4.00 (0.91) | 4.00 (0.67) | 4.21 (0.85) |
| Interoceptive Accuracy | 71.56 (10.05) | 73.28 (13.14) | 73.00 (15.59) | 74.47 (14.26) |
| Interoceptive Attention | 72.25 (10.23) | 70.39 (11.66) | 72.42 (17.42) | 71.85 (15.22) |
| Parental Attachment (female caregiver) | 81.94 (18.04) | 83.82 (15.07) | 84.00 (20.53) | 85.84 (19.95) |
| Parental Attachment (male caregiver) | 89.50 (14.70) | 83.60 (13.03) | 79.24 (20.65) | 77.05 (24.35) |
| Peer Attachment | 85.95 (12.98) | 87.11 (8.98) | 90.11 (13.84) | 93.45 (13.59) |
| GAD-7 | 9.30 (6.85) | 7.62 (7.74) | 8.89 (7.75) | 8.85 (8.35) |
| PHQ-8 | 9.10 (7.25) | 8.38 (8.37) | 8.75 (6.97) | 8.95 (8.74) |
| AUDIT | 0.20 (0.70) | 0.19 (0.87) | 0.00 (0.00) | 0.43 (1.21) |
| DUDIT | 1.10 (4.92) | 0.43 (1.96) | 0.00 (0.00) | 0.57 (2.62) |
| MPVS | 35.17 (6.37) | 38.72 (9.60) | 35.39 (8.26) | 43.44 (13.70) |
| Loneliness | 10.28 (0.89) | 10.33 (1.24) | 10.94 (1.26) | 10.29 (0.99) |
| Self-Perception | NA* | NA* | 8.94 (1.95) | 9.56 (2.34) |

* Note, self-perception questions were only added during the course of the feasibility study and therefore no baseline assessment data was collected for this measure.

**Appendix I: Interview Schedule for Participants**

Introduction, consent form and rapport building

Explain the purpose of the interview to the young person: that we are still trying to make the groups better, and so we would really appreciate their help to make the groups as best as they can be. Only they know what it was like to attend the groups, so they are the experts that can help us. This means we are looking for their honest feedback, including the things they didn’t enjoy as much.

Say that we are going to ask questions from the very beginning of the groups to the very end, so if they can remember things to the best of their ability that would be really helpful for us but this isn’t a memory test so it’s fine if there are things they can’t remember!.

**Screening questionnaire**

I’m going to ask you some questions about the first time you heard about the project which was when we asked you to complete some survey questions in form time. Can you remember that?

1. Can you tell me how you felt about answering those questions in form time and what you understood about what you were being asked to do? [have consent form and survey on hand for reference]

Prompt: Was there anything you didn’t understand about the survey?

Did you read all the information and did you understand everything when you agreed to take part? Was there anything that would have helped you to understand what was being explained?

**Assessments**

I want you to think about the activities we did on the iPads, these were the questions about your feelings, your relationships and your wellbeing. We did these activities twice, once before the group began and once a couple of weeks ago, once the group had finished.

1. How did you feel about answering those questions and completing the activities in a group?

Prompt: if you had the choice of answering these by yourself with a researcher, or in a group with other pupils, would you have a preference and why?

Prompt: Were there any questions you didn’t feel comfortable answering, and would you have felt more comfortable answering them if you were by yourself?

1. (OPTIONAL) What did you think about the amount of time it took to complete the activities?

Prompt: Was it too much, too little, or about the right amount of time?

**Group sessions**

We’re now going to talk about the groups you took part in. Remember, we want you to be as honest as possible, so we want to hear the good as well as the bad

1. How did you find taking part?

Prompt: Did you find the group helpful?

1. In the first meeting you had with your group leader you discussed your goals for taking part in the group. Do you remember the goal you set? You don’t need to tell us what it was. Do you think you made progress towards your goal by the end of the group? [expect a yes/no answer here].
2. Follow up:
3. What do you think helped to make progress?
4. If you didn’t make progress, why didn’t you?
5. What might needed to have been different to help you make progress towards that goal?
6. Was there anything in the group that there any specific skills or strategies that helped you work towards your goals?

1. What did you think about completing the iPad tasks during the sessions?
2. Did you have any difficulties using the iPads?
3. Did you complete the tasks to the best of your ability in each session, or did you feel under pressure to complete them quickly?

1. Thinking about the iPad tasks.. Were you able to use any of the strategies learned from the iPad activities (tuning into yourself, looking for positive possibilities, or reinterpreting/distancing from a scenario) in real-life outside the group?
2. Prompt: can you tell me how you might be able to use each of the strategies in the real world? [can prompt what the tasks were; faces, scenarios, interoception]?

Prompt: Can you say what the situation was, and how the strategy from the iPad activity helped?

1. Were there any other strategies you learned in the groups that you can remember and have used in real-life situations? [maybe ask this question several times until they can no longer name a new strategy]

Prompt: Can you describe the situation where the strategy was helpful, and how you used it?

Prompt: do you still use this strategy? Are there any others you still use?

Prompt if the young people haven’t used the strategies: Is there anything that might have helped you to use the strategies?

1. If you had to describe the group to your friends, how would you explain it?
2. Follow up: Would you recommend it to them? Why/why not?

1. Was there anything that made you not want to take part in the group?

Prompt: What was the best / worst thing about taking part in the group?

Prompt: Did you have any concerns about missing lessons? Did taking part have any effect on your other schoolwork?

1. I now want you to think about the people who led the groups. Those two won’t be running the groups in all schools, so we need to think about what makes a good facilitator in general. What do you think would make a good facilitator?

Prompt: What would help to make you feel comfortable early on in the groups?

Prompt: What is this person like? Is there anything you wish they had done?

1. I want you to think about the meeting you had halfway through the group with your group leader that we invited your parent to.. Do you remember those? Can you tell me how you found those meetings?

Prompt: Did one of your parents attend? If so, what was it like discussing things with your parent? Can you describe a time when you used one of the strategies with a parent or family member?

Prompt if they didn’t attend: We sent your parent/carer some information about the strategies you learnt in the group. Did you talk to your parents/carer about them or what you were doing in the group? (why/why not?) Did you find it helpful talking to them about it if so?

1. Is there anything else you’d like to add about the groups, or anything you’d like to ask me?

End.
